# Understanding Heterosis, Genetic Effects, and Genome Wide Associations for Forage Quantity and Quality Traits in Multi-Cut Pearl Millet

**DOI:** 10.3389/fpls.2021.687859

**Published:** 2021-11-18

**Authors:** Ponnaiah Govintharaj, Marappa Maheswaran, Michael Blümmel, Pichaikannu Sumathi, Anil Kumar Vemula, Abhishek Rathore, Selvanayagam Sivasubramani, Sandip Mallikarjun Kale, Rajeev Kumar Varshney, Shashi Kumar Gupta

**Affiliations:** ^1^Centre for Plant Breeding and Genetics, Tamil Nadu Agricultural University, Coimbatore, India; ^2^International Crops Research Institute for the Semi-Arid Tropics (ICRISAT), Patancheru, India; ^3^International Livestock Research Institute (ILRI), Patancheru, India

**Keywords:** single cross hybrids, top cross hybrids, line × tester, general and specific combining ability, non-additive gene action, association mapping, gene annotation, biomass

## Abstract

Pearl millet is an important food and fodder crop cultivated in the arid and semi-arid regions of Africa and Asia, and is now expanding to other regions for forage purpose. This study was conducted to better understand the forage quantity and quality traits to enhance the feed value of this crop. Two sets of pearl millet hybrids (80 single cross hybrids in Set-I and 50 top cross hybrids in Set-II) along with their parents evaluated multi-locationally for the forage-linked traits under multi-cut (two cuts) system revealed significant variability for the forage traits in the hybrids and parents. The mean better parent heterosis (BPH) for total dry forage yield (TDFY) was 136% across all the single cross hybrids and 57% across all the top cross hybrids. The mean BPH for *in vitro* organic matter digestibility (IVOMD) varied from −11 to 7% in the single cross hybrids and −13 to 11% in the top cross hybrids across cuts. The findings of TDFY and IVOMD heterosis in these sets indicated the potential of improvement of the hybrid cultivars for forage quantity and quality in forage pearl millet. The parental lines single cross parent (SCP)-L02, SCP-L06, and top cross parent (TCP)-T08 found superior in the forage quantity and quality traits can be utilized in the future breeding programs. Most of the forage traits were found to be controlled by using the non-additive gene action. A diverse panel of 105 forage-type hybrid parents (Set-III) genotyped following genotyping by sequencing (GBS) and phenotyped for crude protein (CP) and IVOMD under multi-cuts for 2 years identified one stable significant single nucleotide polymorphism (SNP) on LG4 for CP, and nine SNPs for IVOMD distributed across all the linkage groups except on LG2. The identified loci, once validated, then could be used for the forage quality traits improvement in pearl millet through marker-assisted selection.

## Introduction

Pearl millet [*Pennisetum glaucum* (L) R.Br.] is cultivated mainly in the hot and dry agro-ecologies of Africa and Asia. This crop has a high potential for biomass production due to its C_4_ photosynthetic pathway, and it additionally possesses tolerance to various climatic stresses. Currently, this crop is gaining popularity among the small-holder farmers because of its potential to adapt to the diverse agro-climatic conditions where most of the other crops, such as wheat, rice, sorghum, maize, and barley fail to produce economic yields (Baltensperger, 2002; Vadez et al., 2012).

Pearl millet is primarily cultivated for human consumption in the developing countries. Besides grain production, it is grown as feed and forage crop for livestock grazing, silage, hay, and green fodder chopping (Newman et al., 2010) and is fed to the animals at any crop growth stage without any adverse effect (Arya et al., 2013) in a range of countries, such as the United States (Sheahan, 2014), in the summer season in Australia (Hanna, 1996), Canada (Brunette et al., 2014), Mexico (Urrutia et al., 2015), in triple cropping system in southern Kyushu, Japan (Li et al., 2019), Iran (Aghaalikhani et al., 2008), Central Asia (Nurbekov et al., 2013), and Brazil (Dias-Martins et al., 2018). Recently, it has emerged as an important fodder crop during the summer months of north-western India (Amarender Reddy et al., 2013).

Lack of sufficient quantity of fodder is the major constrain to livestock production in the smaller farming communities in the arid and semi-arid regions. For instance, at present the world has feed shortage of about 911 million tons and would require 1,148 million tons feed by 2030 (Food and Agriculture Organization [FAO], 2002). To alleviate the feed shortage in these regions, further exploitation of pearl millet could be one of the promising solutions as it is well adapted to the arid and semi-arid regions with several other benefits, such as high tillering potential and quick regenerative ability assuring the possibility of multi-cutting, which allows the year-round supply of green/dry forage (Babiker et al., 2014). The results from the multi-location trials conducted during the summer season under the All India Coordinated Forage Project showed pearl millet varieties had higher green forage yield (GFY: 38 t ha^–1^) and crude protein (CP: 9%) in comparison with sorghum (33 t ha^–1^ and 6%) and maize (31 t ha^–1^ and 5.5%) varieties in single cut (Rai et al., 2004). Almost all the released forage cultivars of pearl millet available in the market till date are based on single cut, but milk producing farmers in the semi-arid regions are now demanding multi-cut (2–3 cuts/season) forage with increased digestibility to meet year-round supply of forage/feed. In addition, the pearl millet cultivars bred in the past were dual purpose types with no focus on the improved forage quality, hence were rejected by the farmers because of poor fodder quality of the stover (Kelley et al., 1996). The need has been felt to breed exclusive forage multi-cut type cultivars with better quality to feed the livestock.

The studies based on the animal experiments concluded that the cows fed with pearl millet silage produced milk with increased milk fat concentration than those fed with corn silage (Amer and Mustafa, 2010). Similarly, the cows fed with pearl millet silage consumed more dry matter than those fed with sorghum silage or tropical corn silage (Amer and Mustafa, 2010). Most recently, Lauriault et al. (2021) observed that both pearl millet and sorghum-sudangrass produced equal dry matter yield at single cut forage, and the pearl millet crop provided greater average daily gains than sorghum–sudangrass in beef when both have the same levels of nutritive value. In another livestock performance study by Vinutha et al. (2021), Nellore ram lambs fed with pearl millet silage showed increased digestibility than those fed with sorghum silage harvested at 76 days after sowing. These studies suggest that pearl millet is an excellent choice for the farmers and milch animals in terms of forage yield and quality as compared with other cereals in the drier regions of the world. Hence, targeted breeding efforts have been initiated in last one decade to improve the forage yield and quality traits in pearl millet (Rai et al., 2012; Gupta et al., 2015; Ponnaiah et al., 2019).

Exploitation of heterosis in pearl millet is considered as an easy tool with its protogynous nature of flowering and availability of stable cytoplasmic male sterility (CMS) system (Burton, 1965). Several studies have been conducted to assess the hybrid performance and heterosis in the grain type parents/populations in pearl millet (Pucher et al., 2016; Gupta et al., 2018, 2020; Sattler et al., 2019; Singh and Gupta, 2019; Patil et al., 2020; Sattler and Haussmann, 2020; Dutta et al., 2021). No research has been undertaken to assess the magnitude of heterosis exclusively for the forage yield and quality traits in multi-cut pearl millet.

Limited number of quantitative trait loci (QTL) mapping studies have been conducted in pearl millet for the fodder linked traits, such as for tiller numbers (Varshney et al., 2017), stover yield (Yadav et al., 2002; Nepolean et al., 2006), *in vitro* organic matter digestibility (IVOMD), and nitrogen content (Nepolean et al., 2006). Hash et al. (2003) reported that the QTL mapping and marker-assisted selection (MAS) can be implied for stover yield, forage disease resistance, and for *in vitro* estimates of the nutritive value of several stover fractions of pearl millet for ruminants.

Considering that there is limited information on the gene action, heterosis, and mapping of forage traits under multi-cut system in forage type pearl millet, the present study aimed to estimate heterosis, general combining ability (GCA), and specific combining ability (SCA) for the forage traits of single cross and top cross hybrids at two cutting intervals, and to identify the QTLs for the important forage quality traits in pearl millet.

## Materials and Methods

### Plant Materials

In the present study, two sets of test crosses were developed to investigate heterosis, combining ability, and gene effects for the forage traits. The first set consisted of 80 single cross hybrids (hereafter, referred as Set-I) generated by crossing 10 seed (A-lines) parents and eight pollinator (R-lines) parents having forage type traits (Supplementary Table 1) in a line × tester mating design. The second set comprised of 50 top cross hybrids (hereafter, referred as Set-II) developed by crossing five seed parents (A-lines) and 10 germplasm accessions/open pollinated varieties (OPVs) as pollinators (Supplementary Table 2) in line × tester mating design. These pollinators were already identified as the promising breeding lines for having higher biomass (Gupta et al., 2015).

A third set (Set-III) of 105 diverse hybrid parents comprised of 17 seed and 88 pollinator parents [such as parental lines from Set-I (one pollinator parent not included due to poor quality of DNA from Set-I) and Set-II (excluding pollinators of set-II which were populations)] (Supplementary Table 3) derived from an advanced high biomass nursery (F_6_ and above) of the pearl millet breeding program of the International Crops Research Institute for the Semi-Arid Tropics (ICRISAT), Patancheru, Telangana, India, was used to examine for the genome wide association study (GWAS) for the two forage quality traits CP and IVOMD.

### Evaluation of Parental and Hybrid Trials

Both the single cross (Set-I) and top cross (Set-II) hybrids were evaluated along with the parents and commercial checks at two locations, namely, ICRISAT, Patancheru (18°N, 78°E, and 545 m above sea level) and Tamil Nadu Agricultural University (TNAU), Coimbatore, Tamil Nadu, India (11°N, 77°E, and 411.98 m above sea level) during the summer season of 2015. The hybrids and parents were planted side by side in each trial as separate blocks in each replication to avoid any suppressive effect of the hybrids over parents. The parental block included the fertile (B-lines) lines corresponding to their male sterile (A-lines) lines used in the development of hybrids. Four forage pearl millet commercial hybrids popular in India, namely, PAC 981 (Nutrifeed), DFMH 31, Milkon, and Poshan were used as checks in the hybrids block.

Both the hybrids and parents were planted in alfisol soils in alpha lattice design with three replications at ICRISAT, Patancheru. Each entry was planted in the four rows of 4 m length with rows spaced 60 cm apart and the plants spaced at 10–12 cm from each other. All the hybrids and parents were planted in black soils in alpha lattice design with the two replications at TNAU, Coimbatore. Each entry was planted in the four rows, each of 4 m length and the rows spaced 45 cm apart. The experiment was conducted during first week of March to the third week of June 2015. The trial was irrigated at 12–15 days interval to avoid any moisture stress, and the crop was protected from the weeds, pests, diseases, and animals at both the locations.

The third set (Set-III) of 105 hybrid parents was evaluated at ICRISAT, Patancheru, in alfisol soils during the summer seasons of 2015 and 2016 in partially balanced alpha lattice design with two replications. The plot size, spacing, and field management were same as specified above in the evaluation of first and second sets of trials (of hybrids and parents) at ICRISAT. The experiment was conducted during the first week of March to the third week of June 2015 and 2016.

### Recording of Morphological and Biochemical Traits

In both Set-I and Set-II trials, the four rows of each entry (hybrids and parents) were harvested manually by cutting at the second node from the bottom of the plant at 50–55 days after planting (at boot stage of plants) as the first cut. At the time of harvest, the plant height (PH, centimeters) was measured on five random plants from the base of the stem to the tip of the panicle of the main tiller. Fresh weight of the green forage was recorded (kilogram) for each plot. A sub-sample (10–15 plants) of about 1 kg was collected per entry at the time of harvest and recorded for green forage weight, oven dried for 8 h daily for 3–4 days at 60°C in Campbell dryer (Campbell Industries, Inc., 3201 Dean Avenue, Des Moines, IA, United States), and weighed again (dry forage weight in kilogram). The dry matter concentration and dry forage yield (DFY) of each entry was calculated using the formula:


Dry⁢matter⁢concentration⁢(DMC)=Dry⁢forage⁢weight/Green⁢forage⁢weight×100

DFY=Green⁢forage⁢weight×dry⁢matter⁢concentration

The GFY and DFY were expressed in units of tons per hectare (t ha^–1^). The dried sub-samples of the whole plants (10–15 plants) of each entry were chopped into 10–15 mm pieces using a chaff cutter (Model # 230, Jyoti Ltd. Vadodara-India) and ground using Thomas Wiley mill (Model # 4, Philadephia, PA, United States) to pass through 1-mm screen for the chemical analysis. The ground stover samples (approximately, 40 g of sample/entry) were analyzed by near-infrared reflectance spectroscopy (NIRS) for stover nitrogen concentration (N × 6.25 equals to CP content), neutral detergent fiber (NDF), acid detergent fiber (ADF), acid detergent lignin (ADL), metabolizable energy (ME), and IVOMD as described by Bidinger and Blümmel (2007) and Blümmel et al. (2007). The second cut of forage was harvested from the same four rows (which were subjected to first cut) after 30 days. The forage quantity and quality traits were also recorded in the second cut, as similar to how it was done in the first cut. The total green forage yield (TGFY) and total dry forage yield (TDFY) (t ha^–1^) were estimated as the sum of the two cuts for each entry in this trial.

In case of the Set-III of diverse hybrid parents trial, the same cutting methodology and the measurement of traits (CP and IVOMD) over 2 years of evaluation were followed as similar to that of first and second sets of evaluation trials at ICRISAT.

### DNA Extraction, Genotyping by Sequencing, and Single Nucleotide Polymorphism Calling in the Set-III (Hybrid Parents Panel)

The fresh leaf tissues (100 mg) were collected from 20 to 25 seedlings (per accession) of 8 days old plants (days after sowing) planted in the pots in darkroom at 18°C–25°C, and the DNA was isolated using the NucleoSpin^®^ 96 Plant II kit (Macherey-Nagel, Düren, Germany). Normalization of genomic DNA to 10 ng/μl was done in 0.8% agarose gel using lamda DNA (MBI Fermentas, Hanover, MD, United States). Electrophoresis was performed in Tris acetate-ethylenediaminetetraacetic acid (EDTA) buffer in buffer tank at 90 V for 1 h and the gels were stained with ethidium bromide and visualized in UV light using image analyzer. The DNA of 105 hybrid parents was genotyped using genotyping by sequencing (GBS) (Elshire et al., 2011) by digesting DNA with *Ape*KI endonuclease restriction enzyme. The PCR amplification of pooled amplicons was carried out before sequencing on Illumina Hiseq2500 platform (IlluminaInc, San Diego, CA, United States). The raw sequencing reads and barcode information were processed for single nucleotide polymorphism (SNP) identification from the published pearl millet reference genome (Varshney et al., 2017) using TASSEL v4.0 software (Bradbury et al., 2007). Barcode containing reads were retained and used for SNP calling. These reads were trimmed to 64 bp from barcode side, aligned against each other and used for SNP identification. The identified SNPs were assigned to each hybrid parent based on the information of the barcode sequence. Further, the SNP data were filtered with minor allele frequency (MAF) cut-off of 0.10 (10%) and SNP with ≥ 25% missing data resulted 77,892 SNP markers (Dataset available on ICRISAT Dataverse: http://dataverse.icrisat.org/dataset.xhtml?persistentId=doi%3A10.21421%2FD2%2FIWXCUJ&version=DRAFT). These were further filtered for site coverage (90%), minor allele frequency (0.05), and maximum heterozygosity (50%), were identified 34,691 SNPs and were used for the GWAS analysis.

### Statistical Analysis

The complete parental and hybrids data information were available at first cut of single cross hybrids (10 × 8 = 80 hybrids) and top cross hybrids (5 × 10 = 50 hybrids). In Set-I, the missing data (due to poor regeneration ability of parents and hybrids after first cut) from parents [one line and three testers at one site (TNAU, Coimbatore)] resulted into dropping of 35 single cross hybrids. Hence, 9 × 5 = 45 single cross hybrids were analyzed for second cut in Set-I. Whereas, in Set-II, 26 top cross hybrids were removed from further analysis due to the missing data from parents [one line and four testers at one site (TNAU, Coimbatore)] and thus 4 × 6 = 24 top cross hybrids were analyzed at second cut.

The genotype means were calculated from the combined ANOVA across the two locations using PROC MIXED (SAS Institute Inc, 2017). The estimation of better parent heterosis (BPH) and standard heterosis (SH) were calculated using the formula:


$$\mathrm{BPH}=\left[\left({\text{F}}_{1}-\mathrm{BP}\right)/\mathrm{BP}\right]\times 100,\mathrm{and},$$

$$\text{SH}=\left[\left(F_{1}-\mathrm{CH}\right)/\mathrm{CH}\right]\times 100,$$

where, F_1_ is the hybrid value, BP (better parent) is either P_1_ or P_2_ and CH is the value of the check hybrid. BP was considered to have higher values for PH, GFY, TGFY, DFY, TDFY, CP, ME, and IVOMD, and lower values for NDF, ADF, and ADL. Among the four check hybrids, the PAC 981 (Nutrifeed) is a popular high forage yielding hybrid from the Advanta seed company, which occupies large areas of pearl millet for fodder use in India. Therefore, Nutrifeed was considered as a best check hybrid for calculating the SH in the single cross and top cross hybrids.

The line × tester mating design (Kempthorne, 1957; Arunachalam, 1974) was used to estimate the variance components for Set-I and Set-II hybrids using SAS v9.4 (SAS Institute Inc, 2017). The relative importance of GCA and SCA was also assessed by estimating the components of variance and expressing them in the ratio, 2σ^2^GCA/(2σ^2^GCA + σ^2^SCA) (Baker, 1978). The Pearson’s correlation of hybrid performance with sum of parental GCA effects, SCA effects, and BPH, and also among the BPH of forage traits were determined using GraphPad Prism v5.0 (GraphPad Software, Inc., San Diego, CA, United States).

In GWAS analysis, a set of 105 pearl millet hybrid parents, which were genotyped (34,691 GBS-identified SNPs) and phenotyped (for CP and IVOMD at two cutting intervals) were used for further statistical analysis. The principal component analysis (PCA) was conducted and the resulted eigenvalue (5) was taken into the consideration to correct the population structure along with kinship matrices (K) values implemented in the multi-locus mixed model (MLMM) (Segura et al., 2012) using the R package Genomic Association and Prediction Integrated Tool (GAPIT) (Wang and Zhang, 2020) for GWAS analysis. For population stratification, the Quantile-Quantile (Q-Q) plots were obtained by plots of observed − log10 *P*-value vs. expected − Log10 *P*-value for all the markers. If there is deviation in the *P-*values at initial stage, it indicates the existence of population stratification. Manhattan plots were used to visualize the results of GWAS. Negative Log10 of the *P*-values for each SNPs were plotted over the seven chromosomes for the two traits, CP and IVOMD. The best linear unbiased predictors (BLUPs) were estimated for CP and IVOMD for each hybrid parent across years from the combined analysis (SAS Institute Inc, 2017). The Bonferroni correction threshold was used for selecting significant SNP with an alpha value of 0.05. The significant MTA markers were functionally annotated based on the reference pearl millet genome (v1.1) (Varshney et al., 2017) and its features, using SnpEff software version 4.3t (Cingolani et al., 2012). The gene annotations were further mapped to the homologous functions provided at pfam. Broad sense heritability (*H*^2^) of the traits was estimated by dividing the genotypic variance by the total phenotypic variance (SAS Institute Inc, 2017).


$$H^{2}=\sigma \,g^{2}/\left(\sigma \,g^{2}+\sigma \,g\,e^{2}/l+\sigma \,e^{2}/r\,l\right),$$

where σ*g*^2^ is the genotypic variance, σ*ge*^2^ is the genotype × environment variance, σ*e*^2^ is the error variance, r represents the replicates, and “l” represents the locations.

## Results and Discussion

### Variance Analysis for Forage Traits

Combined ANOVA exhibited significant differences for most of the forage traits among the genotypes except TDFY, CP, and IVOMD at second cut in the top cross hybrids (Supplementary Table 4), indicating considerable variation among the parents and hybrids for majority of the forage traits. The location × hybrids interaction effect was significant for TDFY in the single cross hybrids, CP at first cut in both the single cross and top cross hybrids, and IVOMD at second cut in the single cross hybrids and at first cut in top cross hybrids, suggesting that the hybrids need to be evaluated under multiple sites and multiple years/seasons to identify stable cultivar. In line with the current results, Shinde (2011) reported high location × hybrids interaction effects for CP and IVDMD (*in vitro* dry matter digestibility) in pearl millet.

### Mean Performance of Hybrids and Heterosis for Forage Traits

The mean TDFY (15 t ha^–1^) in the single cross hybrids was found to be higher than those of the top cross hybrids (TDFY: 11 t ha^–1^) (Table 1), suggesting single cross hybrids offers opportunities for the increased forage productivity than the top cross hybrids. The BPH for TDFY ranged from 13 to 422% with a mean of 136% in single cross hybrids and 0 to 176% with a mean of 57% in the top cross hybrids (Table 2), indicating great potential for increasing the forage productivity in pearl millet. The results showed that 71 of 80 single cross hybrids (89%) and 25 of 50 top cross hybrids (50%) had greater than 35% TDFY than the better parent (Supplementary Tables 5, 6), indicating overdominance gene action plays a major role for TDFY under study. The results obtained for BPH of forage yield in this study were higher than the BPH of forage yield reported earlier in the single cross hybrids (Gupta et al., 2018; Ponnaiah et al., 2019) and in top cross or population hybrids (Bidinger et al., 2003; Pucher et al., 2016; Sattler et al., 2019) of pearl millet. Fifteen and 30 single cross and top cross hybrids significantly outyielded the check hybrid Nutrifeed by ≥15% for TDFY, respectively (Supplementary Tables 7, 8).

**TABLE 1:** The mean performances for the forage related morphological and biochemical traits of both the single cross hybrids (SCHs) and top cross hybrids (TCHs) for both the cuts, evaluated summer season of 2015 at International Crops Research Institute for the Semi-Arid Tropics (ICRISAT), Patancheru, and Tamil Nadu Agricultural University (TNAU), Coimbatore.

Traits	Single cross												Top cross											
	Parents						Hybrids						Parents						Hybrids					
	Minimum		Maximum		Average		Minimum		Maximum		Average		Minimum		Maximum		Average		Minimum		Maximum		Average	
	*FC	*SC	FC	SC	FC	SC	FC	SC	FC	SC	FC	SC	FC	SC	FC	SC	FC	SC	FC	SC	FC	SC	FC	SC
**Desirable traits**																								
PH (cm)	NA	51.8	NA	104.7	NA	81.6	NA	94.6	NA	152.1	NA	125.8	93.9	70.5	177.0	93.3	131.4	81.1	144.9	100.9	212.0	139.9	188.9	119.3
GFY (t ha^–1^)	12.3	0.7	35.4	5.7	26.5	2.8	30.9	1.9	55.6	9.1	42.5	5.7	21.4	0.5	63.4	5.3	44.2	2.7	31.5	2.3	50.8	5.7	41.3	4.1
TGFY (t ha^–1^)	13.6		38.8		28.0		34.0		60.4		47.6		23.0		66.2		46.7		33.8		55.0		45.1	
TDFY (t ha^–1^)	3.2		8.7		5.4		6.8		25.1		14.5		2.5		8.9		6.2		6.4		21.8		10.8	
DFY (t ha^–1^)	3.2	0.3	10.4	1.8	5.0	0.9	8.3	0.7	21.7	2.7	14.2	1.7	3.0	0.2	8.2	1.7	5.9	0.9	8.2	0.8	19.5	1.8	11.3	1.2
CP (%)	10.8	9.6	14.6	12.9	12.7	11.4	9.4	8.6	13.7	12.0	11.4	10.4	10.4	10.1	13.7	12.4	11.8	11.3	9.0	9.2	12.5	11.2	10.8	10.0
ME (MJ kg^–1^)	6.5	6.3	7.2	7.2	6.8	6.8	6.3	6.5	7.6	7.3	7.1	6.9	6.2	6.7	6.9	7.1	6.6	6.9	6.0	6.6	7.5	7.4	6.8	6.9
IVOMD (%)	47.9	46.4	52.9	52.8	49.9	49.5	45.4	47.1	54.2	52.4	50.8	49.8	46.9	48.2	50.4	51.9	48.3	50.2	43.8	48.1	53.3	53.2	49.0	50.0
**Undesirable traits**																								
NDF (%)	61.7	61.5	68.2	65.9	64.8	63.5	62.9	63.3	68.5	67.5	65.7	64.9	65.2	62.0	69.6	66.1	67.2	63.6	65.3	63.8	72.4	66.5	68.1	65.5
ADF (%)	28.4	28.0	32.5	32.4	30.9	30.5	27.7	29.8	33.7	34.3	30.7	31.9	31.7	29.1	35.1	33.3	33.3	31.2	30.0	29.3	38.3	33.2	33.3	31.8
ADL (%)	3.6	3.7	4.6	4.4	4.2	4.0	3.6	3.8	5.1	4.7	4.3	4.2	4.4	3.5	5.0	4.2	4.7	3.9	4.0	4.0	5.7	4.4	4.7	4.2

*FC: first cut; SC: second cut. PH: plant height; GFY: green forage yield; TGFY: total green forage yield; DFY: dry forage yield; TDFY: total dry forage yield; CP: crude protein; NDF: neutral detergent fiber; ADF: acid detergent fiber; ADL: acid detergent lignin; ME: metabolizable energy; IVOMD: in vitro organic matter digestibility.

**TABLE 2:** Heterosis for the forage linked morphological and biochemical traits in SCHs and TCHs evaluated in summer season of 2015 at ICRISAT, Patancheru, and TNAU, Coimbatore.

Traits	Materials	Cutting intervals	Better-parent heterosis (%)			Standard heterosis (%)		
			Minimum	Maximum	Average	Minimum	Maximum	Number of hybrids better than check
**Desirable traits**								
PH (cm)	^‡^SCHs	First cut (80)a	*NA*	*NA*	*NA*	–43.8	–1.5	0
		Second cut (45)b	4.0	73.2	37.3	–27.5	17.5	25
	^†^TCHs	First cut (50)c	2.3	90.1	35.1	–20.8	2.4	2
		Second cut (24)d	15.4	77.9	41.4	–17.1	17.7	21
GFY (t ha^–1^)	SCHs	First cut	–8.2	74.4	32.6	–51.5	10.5	5
		Second cut	–37.0	301.9	51.5	–44.8	27.5	22
	TCHs	First cut	–46.1	2.9	–20.2	–27.3	12.7	6
		Second cut	–57.7	101.4	26.1	–62.8	14.2	7
TGFY (t ha^–1^)	SCHs	Combined	1.8	118.4	47.9	–39.3	8.0	7
	TCHs	combined	–40.7	5.3	–18.1	–27.6	10.2	7
TDFY (t ha^–1^)	SCHs	Combined	13.3	421.9	136.0	–61.8	35.2	9
	TCHs	combined	0.0	176.1	56.5	–48.1	75.8	10
DFY (t ha^–1^)	SCHs	First cut	–13.1	344.3	154.2	–68.2	–0.9	0
		Second cut	–29.7	248.2	50.7	–37.7	11.2	9
	TCHs	First cut	16.9	318.7	75.5	–33.9	9.1	3
		Second cut	–55.1	109.3	19.5	–44.4	7.3	4
CP (%)	SCHs	First cut	–27.8	8.8	–14.4	–11.2	9.9	15
		Second cut	–30.7	4.5	–12.8	–14.0	8.8	12
	TCHs	First cut	–33.0	0.3	–15.2	–12.2	3.0	2
		Second cut	–23.2	–3.1	–14.7	–9.2	9.4	15
ME (MJ kg^–1^)	SCHs	First cut	–6.1	7.7	1.7	–2	1.6	18
		Second cut	–8.5	3.9	–2.1	–4.1	2.3	24
	TCHs	First cut	–12.1	12.5	1.6	–2.5	1.1	14
		Second cut	–6.6	4.9	–0.8	–2.5	2.9	2
IVOMD (%)	SCHs	First cut	–8.2	6.7	0.1	–3.4	2.6	12
		Second cut	–10.8	3.4	–2.9	–2.9	1.6	21
	TCHs	First cut	–13.2	11.3	0.4	–1.2	0.6	19
		Second cut	–6.1	3.6	–1.8	–2.5	3.1	6
**Undesirable traits**								
NDF (%)	SCHs	First cut	–7.8	6.4	–0.4	–2.8	1.4	47
		Second cut	–3.9	7.3	1.2	–1.8	2.2	28
	TCHs	First cut	–4.5	7.6	0.2	–1.6	3.1	13
		Second cut	–1.7	6.3	2.0	–2.4	2.8	16
ADF (%)	SCHs	First cut	–13.2	3.7	–3.1	–4.2	2.6	26
		Second cut	–7.5	14.3	2.3	–1.0	2.4	20
	TCHs	First cut	–11.3	15.3	–1.5	–0.1	3.4	2
		Second cut	–10.6	6.3	–0.2	–1.3	2.2	20
ADL (%)	SCHs	First cut	–20.2	14.0	–1.9	–6.9	8.0	9
		Second cut	–12.0	19.1	2.4	–1.2	6.8	10
	TCHs	First cut	–17.3	18.5	–2.0	–2.4	14.2	4
		Second cut	–6.6	18.3	3.9	–1.3	7.5	3

*a, b, c, and d indicates 80, 45, 50, and 24 hybrids, respectively; ^‡^ SCHs: single cross hybrids;*

*^†^ TCHs: top cross hybrids. PH: plant height; GFY: green forage yield; TGFY: total green forage yield; DFY: dry forage yield; TDFY: total dry forage yield; CP: crude protein; NDF: neutral detergent fiber; ADF: acid detergent fiber; ADL: acid detergent lignin; ME: metabolizable energy; IVOMD: in vitro organic matter digestibility.*

Similarly, the forage quality trait CP ranged from 9 to 14% in single cross hybrids and 9 to 13% in top cross hybrids across cuts, which is still higher than the minimum requirement (about 7%) recommended for feed protein in microbes in the rumen of ruminants (Van Soest, 1994). The BPH for CP varied from −31 to 9% and −33 to 0.3% in single cross and top cross hybrids across cuts, respectively. Five (two and three at first and second cuts, respectively) single cross hybrids and one (at second cut) top cross hybrid exhibited numerically positive BPH for CP, but none were significant. The check hybrid Nutrifeed had CP of 11.9% and 10.9% in single cross hybrids and 11.5% and 10.5% in top cross hybrids, at first and second cuts, respectively. Four and one single cross and top cross hybrids across cuts found to have significant positive or numerically higher/or at par with the check hybrid for CP, respectively. The forage IVOMD varied from 45 to 54% and 44 to 53% across cuts in single cross and top cross hybrids, respectively. It has been reported that 1% increase in digestibility (IVOMD) in sorghum and pearl millet stovers could lead to increase in the animal outputs by 6% to 8% (Kristjanson and Zerbini, 1999). The BPH for IVOMD varied from −11 to 7% in single cross hybrids and −13 to 11% in top cross hybrids across cuts. Five single cross hybrids and three top cross hybrids exhibited numerically positive BPH for IVOMD across cuts, with one top cross hybrid significantly greater than BP. However, five single cross hybrids and three top cross hybrids had significant positive or positive BPH combined for TDFY and IVOMD for both the cuts (Supplementary Tables 5, 6). The check hybrid Nutrifeed had IVOMD of 51% and 48.6% at first cut and, 49.9% and 50.5% at second cut, for single cross and top cross hybrids, respectively. Ten single cross hybrids and three top cross hybrids across cuts outperformed the check hybrid (Nutrifeed) for IVOMD values.

The single cross hybrids found to have higher mean values of desirable forage traits, such as TGFY, TDFY, and IVOMD, and low negative mean values of undesirable forage traits, such as NDF, ADF, and ADL at first cut than top cross hybrids (Table 1). This might be due to the involvement of inbreds as parents in single cross hybrids, as inbreds (hybrid parental lines) were bred for the improved forage traits. In addition, the inbred lines have favorable alleles fixed, whereas the alleles in the germplasm/OPVs are having intermediate frequencies (alleles that are not fixed), thereby the genes in top cross hybrids are less likely to combine for the favorable traits to the extent as in case of single cross hybrids (Miranda Filho, 1999; Riday et al., 2002).

Some hybrids from both the single cross and top cross hybrids had higher values for 2–3 forage traits, than the best check hybrid Nutrifeed. For instance, one single cross hybrid (SCH38: L05 × T16) had significant positive standard heterosis (SH) for 12% TDFY, 4% CP and had superior or on par IVOMD with the check hybrid Nutrifeed at first cut (Supplementary Table 7). Besides these, two hybrids SCH12: L02 × T14 and SCH62: L08 × T16 had significant positive or positive SH for TDFY (25% and 12%, respectively) with comparable IVOMD across cuts, and a hybrid SCH17: L03 × T11 had significant positive SH of 15% TDFY and comparable IVOMD at second cut. Similarly, one top cross hybrid (TCH42: L05 × T07) had significant positive or positive SH for 57% TDFY, 9% CP at second cut, and had superior or on par IVOMD with the check hybrid Nutrifeed across cuts, and a hybrid TCH45: L05 × T10 had SH of 37% TDFY and 3% IVOMD at second cut over the check hybrid (Supplementary Table 8). Additionally, two each of single cross (64% and 22% TDFY; 5% and 4% CP at first and second cuts, respectively) and top cross hybrids (57% and 18% TDFY; 10% and 5% CP at second cut) out yielded the check hybrid Nutrifeed for these forage traits. The hybrids identified from single cross and top cross hybrids indicated that it is possible to breed the hybrids for high forage yield or for superior forage quality or combination of both forage yield and better quality in pearl millet.

### Combining Ability and Gene Action

The mean squares due to GCA and SCA variances were found to be significant for TDFY in single cross hybrids, CP at first cut in single cross hybrids, and IVOMD at second cut in single cross hybrids, and also at first cut in top cross hybrids, indicating the importance of both the additive and non-additive effects for these traits (Supplementary Table 4). Highly significant interactions between location × (line × tester) for TDFY in single cross hybrids, CP at first cut in both single cross and top cross hybrids, and IVOMD at second cut in single cross hybrids, and at first cut in top cross hybrids indicted that hybrid performance was influenced by the locations. Baker predictability ratio (PR) was relatively lower to unity (PR ≤ 0.80) for TDFY in both the single cross and top cross hybrids, CP at first cut in single cross hybrids, and for both the cuts in top cross hybrids, and IVOMD for both the cuts in single cross and top cross hybrids, indicating greater importance of the non-additive gene action for important forage traits under study (Table 3 and Supplementary Table 4). These results are in agreement with the findings of Shinde (2011) for CP in single cross hybrids, and Ouendeba et al. (1996) for IVDMD in population hybrids, who reported that forage quality traits were under non-additive gene action in pearl millet. However, TGFY for both the single cross and top cross hybrids, and CP in single cross hybrids of second cut, were controlled by the additive gene action and thus can be improved through selection.

**TABLE 3:** Gene effects for important forage linked morphological and biochemical traits in single cross and top cross hybrids.

Traits	Additive				Non additive			
	Single cross hybrids		Top cross hybrids		Single cross hybrids		Top cross hybrids	
	First cut	Second cut	First cut	Second cut	First cut	Second cut	First cut	Second cut
TGFY (t ha^–1^)	√^†^		√					
TDFY (t ha^–1^)					√		√	
CP (%)		√			√		√	√
IVOMD (%)					√	√	√	√

*√^†^ indicates presence of gene action. TGFY-total green forage yield, TDFY-total dry forage yield, CP-crude protein and IVOMD-in vitro organic matter digestibility.*

The GCA effects of the single cross and top cross parents for the forage quantity and quality traits under different cutting intervals are given in Supplementary Table 9. Seven parents (for TGFY and/or TDFY) in single cross hybrids and three parents (for TDFY) in top cross hybrids had significant positive GCA effects for these forage quantity traits, indicating that these parents can be used to enhance the forage yield potential in breeding high biomass pearl millet. Furthermore, some parents had significant positive GCA effects for multiple forage linked traits: single cross parent (SCP)-L02 (TGFY and IVOMD at second cut), SCP-L06 (TDFY and IVOMD at first cut), and SCP-L10 (TGFY and CP at first cut) in single cross hybrids, and top cross parent (TCP)-T08 (TDFY and IVOMD at first cut) in top cross hybrids. These parents identified as good general combiners can be utilized in the breeding programs aiming to improve the forage traits in the terms of higher forage productivity coupled with superior forage quality under multi-cut system.

The estimates of SCA effects of the single cross and top cross hybrids for the forage quantity and biochemical quality traits are provided in Supplementary Tables 10, 11. Nineteen single cross hybrids and eight top cross hybrids were found to have highly significant positive SCA effects for the forage yield, and thus could be used in the development of high yielding forage hybrids in pearl millet. Additionally, some of the experimental hybrids had significant positive SCA effects for two or more forage traits, for instance, the hybrids (SCH05: L01 × T15 and SCH12: L02 × T14) for forage traits (TDFY and CP at first cut, and TDFY and IVOMD at second cut, respectively) along with high mean values for these traits. Similarly, in top cross hybrids, the hybrids (TCH18: L02 × T13 and TCH23: L03 × T08) at first cut and one hybrid (TCH45: L05 × T10) at second cut had significant positive SCA effects with high mean values for forage traits (TDFY and IVOMD). The identified single cross and top cross hybrids in the present study can greatly contribute to the forage productivity in the terms of forage quantity (TDFY) and quality (CP and IVOMD).

### Correlation Between Heterosis and Combining Ability of Forage Traits

The SCA effects showed significant positive correlations with hybrid performance and BPH for most of the forage traits across cuts in the single cross and top cross hybrids (Table 4). The GCA effects also showed positive significant correlations with hybrid performance for most of the forage traits, but low or even no correlations with BPH (Table 4). These results indicated that both the additive and non-additive gene effects were important for hybrid performance, while the non-additive gene effects were the major cause for heterosis. Similar findings were also reported earlier in other crops, such as in maize (Yu et al., 2020), barley (Bernhard et al., 2017) and in rice (Huang et al., 2015).

**TABLE 4:** Associations among the hybrid performance, better-parent heterosis (BPH), and combining ability in single cross and top cross pearl millet hybrids for the forage linked traits.

Traits	F_1_ vs. GCAsum				F_1_ vs. SCA				GCA vs. BPH				SCA vs. BPH			
	Single cross hybrids		Top cross hybrids		Single cross hybrids		Top cross hybrids		Single cross hybrids		Top cross hybrids		Single cross hybrids		Top cross hybrids	
	First cut	Second cut	First cut	Second cut	First cut	Second cut	First cut	Second cut	First cut	Second cut	First cut	Second cut	First cut	Second cut	First cut	Second cut
PH (cm)	NA	0.78***	0.60***	0.64***	NA	0.62***	0.80***	0.77***	NA	0.09	0.10	0.61**	NA	0.58***	0.42***	0.65***
GFY (t ha^–1^)	0.82***	0.55***	0.62***	0.75***	0.57***	0.83***	0.79***	0.66***	0.43***	–0.41**	0.09	0.51*	0.53***	0.56***	0.67***	0.51*
TGFY (t ha^–1^)	0.63***		0.62***		0.71***		0.78***		0.10		0.15		0.67***		0.70***	
DFY (t ha^–1^)	0.48***	0.72***	0.56***	0.65***	0.88***	0.70***	0.83***	0.76***	0.30**	–0.33*	0.35*	−0.10	0.63***	0.63***	0.52***	0.60**
TDFY (t ha^–1^)	0.33**		0.61***		0.95***		0.78***		0.00		0.38**		0.71***		0.72***	
CP (%)	0.60***	0.73***	0.53***	0.69***	0.80***	0.69***	0.85***	0.73***	–0.12	0.33*	0.26	0.42*	0.67***	0.57***	0.66***	0.58***
NDF (%)	0.55***	0.57***	0.56***	0.55**	0.84***	0.82***	0.82***	0.84**	0.09	0.36*	0.45**	0.23	0.50***	0.64***	0.80***	0.55**
ADF (%)	0.59***	0.59***	0.63***	0.47*	0.81***	0.81***	0.78***	0.72***	–0.16	0.58***	0.55***	0.40	0.71***	0.40**	0.74***	0.60**
ADL (%)	0.59***	0.67***	0.56***	0.66***	0.80***	0.74***	0.83***	0.75***	–0.13	0.46**	0.46***	0.59**	0.69***	0.62***	0.75***	0.38
ME (MJ/kg)	0.51***	0.68***	0.65***	0.60**	0.83***	0.73***	0.76***	0.80***	–0.01	0.34*	0.67***	0.47*	0.84***	0.72***	0.64***	0.68***
IVOMD (%)	0.58***	0.67***	0.60***	0.46*	0.81***	0.74***	0.80***	0.80***	–0.08	0.35*	0.52***	0.27	0.79***	0.52***	0.72***	0.63***

*P < 0.05,

**P < 0.01,

***P < 0.001, and NA: not available. F_1_: hybrid performance, GCAsum: the sum of GCA for two parents, SCA: specific combining ability, BPH: better parent heterosis. PH: plant height; GFY: green forage yield; TGFY: total green forage yield; DFY: dry forage yield; TDFY: total dry forage yield; CP: crude protein; NDF: neutral detergent fiber; ADF: acid detergent fiber; ADL: acid detergent lignin; ME: metabolizable energy; IVOMD: in vitro organic matter digestibility.

Better parent heterosis (BPH) of forage quantity (TGFY and TDFY) traits did not show correlation with the forage quality (CP and IVOMD) traits across cuts in the single cross and top cross hybrids (Supplementary Table 12), indicating that the forage quantity and quality traits can be improved independent of each other. These results are in agreement with the previous studies in pearl millet (Govintharaj et al., 2018) and in other crop, such as in sorghum (Aruna et al., 2015) which reported no associations between the forage quantity and quality traits.

### Genome Wide Associations for the Forage Quality Traits

**FIGURE 1:**
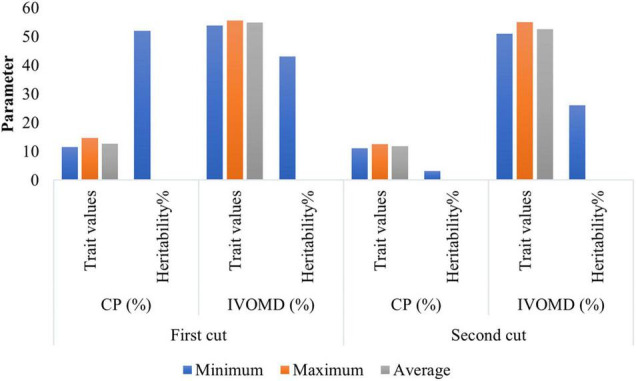
Phenotypic performance of hybrid parents for the forage quality traits in 105 hybrid parents (Set-III) during summer seasons of 2015 and 2016 at International Crops Research Institute for the Semi-Arid Tropics (ICRISAT), Patancheru.

The minimum, maximum, and average values of the two traits, CP and IVOMD, observed at the different cutting intervals over 2 years are shown in Figure 1. The mean CP at first cut was almost on par with the second cut, whereas IVOMD differed by two units between the first and second cuts. The results showed huge variations between the parental lines for CP (11.5%–14.6% at first cut and 11%–12.5% at second cut) and IVOMD (53.8%–55.6% and 51%–55% at first and second cuts, respectively) as compared with the earlier studies in pearl millet (Bidinger et al., 2010; Blümmel et al., 2010). Low to moderate broad sense heritability was observed for CP and IVOMD across the two cuts (Figure 1). However, several other workers (Hash et al., 2006; Bidinger and Blümmel, 2007; Rai et al., 2012; Govintharaj et al., 2017) earlier reported low heritability for the forage quality traits in pearl millet.

**FIGURE 2:**
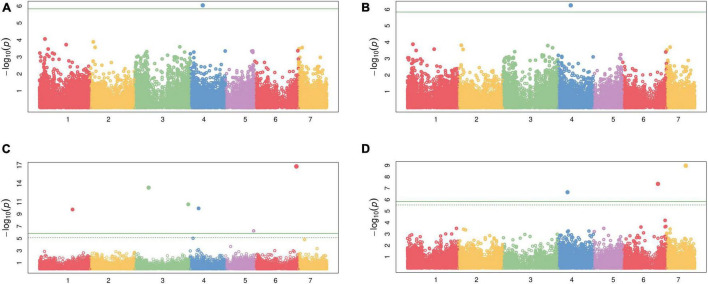
Manhattan plot for the two forage quality traits using 105 diverse pearl millet hybrid parents (Set-III); **(A)** Crude protein (CP) at second cut for 2 years in combined analysis, **(B)** CP at second cut during summer season of 2016, **(C)** *In vitro* organic matter digestibility (IVOMD) at first cut during summer season 2015, and **(D)** IVOMD at second cut during summer seasons of 2016.

Genome wide association study among 34,691 SNPs and each of the two traits, CP and IVOMD, at two different cutting intervals, detected 10 SNPs which were above the chosen threshold level shown in the Manhatton plots (Figure 2 and Table 5), and the corresponding Q-Q plots are provided in the (Supplementary Figure 1). At second cut, during the summer season of 2016, a SNP (S4_69014036) was located on the linkage group 4 (LG4) which was found to be significantly associated with CP with the *p* value of 5.63E-07. The same SNP was found to be tightly associated with CP with the *p* value of 9.05E-07 when analyzed using the pooled hybrid parental data from summer seasons of 2015 and 2016. Six SNPs, one (S6_227902580) on LG6, two (S3_75463586 and S3_291119752) on LG3, and one each on LG4 (S4_46289498), LG1 (S1_180007567) and LG5 (S5_154075820), were found to be significantly associated with IVOMD at first cut during the summer season of 2015, having the *p* values of 1.57E-17, 4.71E-14, 2.53E-11, 1.16E-10, 1.78E-10, and 5.59E-07, respectively. Also, at second cut, during summer season of 2016, three SNPs S7_104645663 (LG7), S6_192886095 (LG6), and S4_51491754 (LG4) with the *p* values of 1.06E-09, 4.16E-08, and 2.21E-07, respectively, were tightly linked with IVOMD. Similar to this study, Nepolean et al. (2006) found SSRs markers linked to the traits CP on LG4, and IVOMD on LG1, and LG6 using the bi-parental mapping population with drought tolerance background in pearl millet. No significant SNPs were found which are common for both CP and IVOMD, in both the cuts, suggesting that the genetic basis of these traits may not be the same (at different cutting intervals). These identified SNPs for the forage quality traits should be validated and then can be introgressed into the genetic background of elite/locally well adapted popular varieties to improve the forage quality in pearl millet through the marker-assisted selection.

**TABLE 5:** The traits linked markers, genes, and their functions identified for the forage quality traits in 105 hybrid parents evaluated during the summer seasons of 2015 and 2016 at ICRISAT, Patancheru.

S.No.	GWAS						Gene annotation			
	SNP	Chr.	Pos.	P.value	Minor allele frequency	FDR_Adjusted_ P.values	Gene	Annotation	Gene function	Gene function retrieved from Genome server
**CP (crude protein) at second cut in combined analysis**										
1	S4_69014036	4	69014036	9.05E-07	0.242857	0.03141	Pgl_GLEAN_10029543	upstream_gene_variant	Mre11 DNA-binding presumed domain	Monocot Plaza and Phytozome
**CP at second cut during summer season of 2016**										
1	S4_69014036	4	69014036	5.63E-07	0.242857	0.01953	Pgl_GLEAN_10029543	upstream_gene_variant	Mre11 DNA-binding presumed domain	Monocot Plaza and Phytozome
***In vitro* organic matter digestibility (IVOMD) at first cut during summer season of 2015**										
1	S6_227902580	6	227902580	1.57E-17	0.147619	5.46E-13	-	intergenic_region	NA	Monocot Plaza and Phytozome
2	S3_75463586	3	75463586	4.71E-14	0.138095	8.17E-10	-	intergenic_region	NA	Monocot Plaza and Phytozome
3	S3_291119752	3	291119752	2.53E-11	0.128571	2.92E-07	Pgl_GLEAN_10024973	synonymous_variant	Coatomer epsilon subunit	Monocot Plaza and Phytozome
4	S4_46289498	4	46289498	1.16E-10	0.095238	1.01E-06	Pgl_GLEAN_10027418	upstream_gene_variant	NHL domain-containing protein	Monocot Plaza and Phytozome
5	S1_180007567	1	180007567	1.78E-10	0.190476	1.24E-06	-	intergenic_region	NA	Monocot Plaza and Phytozome
6	S5_154075820	5	154075820	5.59E-07	0.114286	0.00323	Pgl_GLEAN_10037615	upstream_gene_variant	CCT motif	
**IVOMD at second cut during summer season of 2016**										
1	S7_104645663	7	104645663	1.06E-09	0.390476	3.69E-05	Pgl_GLEAN_10012611	intron_variant	Methyltransferase domain/Hen1 La-motif C-terminal domain	Monocot Plaza and Phytozome
2	S6_192886095	6	192886095	4.16E-08	0.114286	0.00072	Pgl_GLEAN_10034437	synonymous_variant	Coatomer beta C-terminal region	Monocot Plaza and Phytozome
3	S4_51491754	4	51491754	2.21E-07	0.414286	0.00256	Pgl_GLEAN_10032186	splice_region_variant and intron_variant	NA	Monocot Plaza and Phytozome

*Chr.: chromosome; Pos.: position; NA: not available.*

### Gene Annotation for the Forage Quality Traits

One SNP was found closely associated with CP and nine SNPs were found associated with IVOMD (Table 5). The SNP for CP corresponded to the gene Pgl_GLEAN_10029543 that coded for Mre11 DNA-binding presumed domain. Similarly, out of nine SNPs found associated with IVOMD, three were found in coding sequences (CDS) region and one in splice region which were uncharacterized for any of the gene function. The genes Pgl_GLEAN_10034437, Pgl_GLEAN_10012611, Pgl_GLEAN_10027418, Pgl_GLEAN_ 10024973, and Pgl_GLEAN_10037615 were found coding for functions, such as Coatomer beta C-terminal region, Methyltransferase domain/Hen1 La-motif C-terminal domain, NHL domain containing protein, Coatomer epsilon subunit, and CCT motif, respectively. Furthermore, some of the genes, such as Pgl_GLEAN_10029543, Pgl_GLEAN_10034437, Pgl_GLEAN_10012611, Pgl_GLEAN_10027418, Pgl_GLEAN_ 10024973, Pgl_GLEAN_10037615, and Pgl_GLEAN_10032186 were also responsible for cold tolerance (Meng et al., 2021).

## Conclusion

Both the single cross and top cross hybrids had wide variability for the forage linked traits across cuts. The single cross hybrids had higher forage yielding traits (TGFY and TDFY), and higher IVOMD (desirable forage quality trait) and lower NDF, ADF, and ADL (undesirable forage quality traits) at first cut than top cross hybrids. The mean BPH was higher for TGFY, TDFY, and CP across cuts in single cross hybrids than top cross hybrids. Some single and top cross hybrids outperformed the commercial check hybrid for the forage yield and quality traits, these identified hybrids can be further evaluated in the multi-location trials to confirm their yield potential and stability prior to commercial release. The present study has identified the potential lines and testers for GCA effects, combined for both the forage yield and quality traits, which can offer opportunities for developing hybrids with increased forage productivity in pearl millet. Most of the forage traits across cuts in both the single cross and top cross hybrids were predominantly controlled by the non-additive gene action. No significant correlation was observed between the forage quantity and quality traits indicating that these traits can be improved independently. GWAS identified ten genomic regions associated with the forage quality traits (CP and IVOMD), and thus can be further validated, for improving the pearl millet forage quality traits through marker assisted selection. Significant genomic loci and candidate genes identified from this study lay a foundation for the development of high biomass cultivars with superior forage quality trait in the future forage breeding programs.

## Data Availability Statement

The datasets presented in this study can be found in online repositories. The names of the repository/repositories and accession number(s) can be found below: http://dataverse.icrisat.org/dataset.xhtml?persistentId=doi:10.21421/D2/IWXCUJ.

## Author Contributions

SG, PG, MM, PS, and MB designed the experiments. AV, SK, AR, SS, and RV helped in data analysis. RV involved in genotyping for forage type hybrid parents. MB involved in phenotyping for forage quality traits by NIRS. PG and SK wrote the manuscript. MM and PS reviewed the manuscript. All authors read and approved the final manuscript.

## Conflict of Interest

The authors declare that the research was conducted in the absence of any commercial or financial relationships that could be construed as a potential conflict of interest. The handling editor declared a past collaboration with one of the authors, RV.

## Publisher’s Note

All claims expressed in this article are solely those of the authors and do not necessarily represent those of their affiliated organizations, or those of the publisher, the editors and the reviewers. Any product that may be evaluated in this article, or claim that may be made by its manufacturer, is not guaranteed or endorsed by the publisher.

## Abbreviations

SCH: single cross hybrid
TCH: top cross hybrid
SCP: single cross parent
TCP: top cross parent
PH: plant height
GFY: green forage yield
TGFY: total green forage yield
DFY: dry forage yield
TDFY: total dry forage yield
NIRS: near-infrared reflectance spectroscopy
SCP: crude protein
NDF: neutral detergent fiber
ADF: acid detergent fiber
ADL: acid detergent lignin
ME: metabolizable energy
IVOMD: *in vitro* organic matter digestibility
FC: first cut
SC: second cut
BP: better parent
BPH: better parent heterosis
CH: check hybrid
SH: standard heterosis
OPV: open pollinated variety
SCA: specific combining ability
GCA: general combining ability
GBS: genotyping by sequencing
PCR: polymerase chain reaction
MAF: minor allele frequency
SNP: single nucleotide polymorphism
PCA: principal component analysis
BLUP: best linear unbiased predictors
TASSEL, Trait Analysis by aSSociation: Evolution and Linkage
GAPIT: Genome Association and Prediction Integrated Tool
MLMM: multi-locus mixed model
QQ: quantile-quantile
MAS: marker assisted selection
QTL: quantitative trait loci
CMS: cytoplasmic male sterility
IVDMD: *in vitro* dry matter digestibility
ICRISAT: International Crops Research Institute for the Semi-Arid Tropics
TNAU: Tamil Nadu Agricultural University
PR: predictability ratio
ANOVA: analysis of variance.
