# Supplementary material for: Understanding Heterosis, Genetic Effects, and Genome Wide Associations for Forage Quantity and Quality Traits in Multi-Cut Pearl Millet
Source: Front Plant Sci. 2021 Nov 18;12:687859. doi: 10.3389/fpls.2021.687859 (PMC8636684; doi:10.3389/fpls.2021.687859)
Supplement: Supplementary file 1 [file Image_1.pdf]

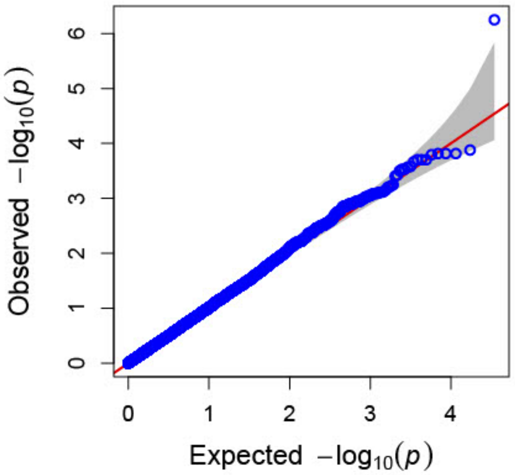

(A)

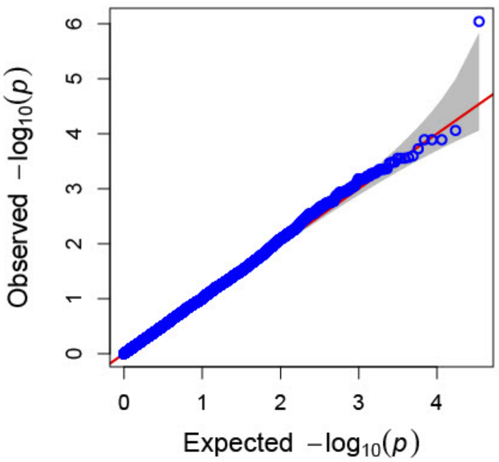

(B)

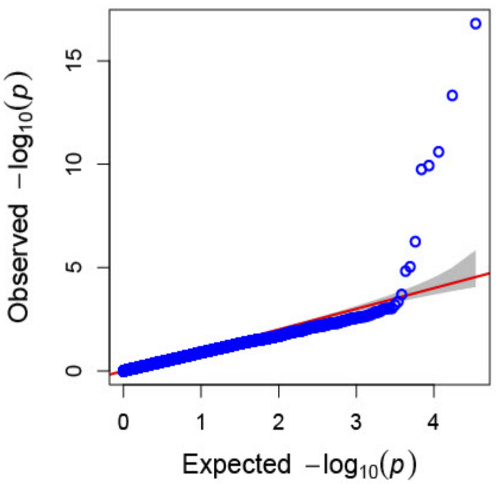

(C)

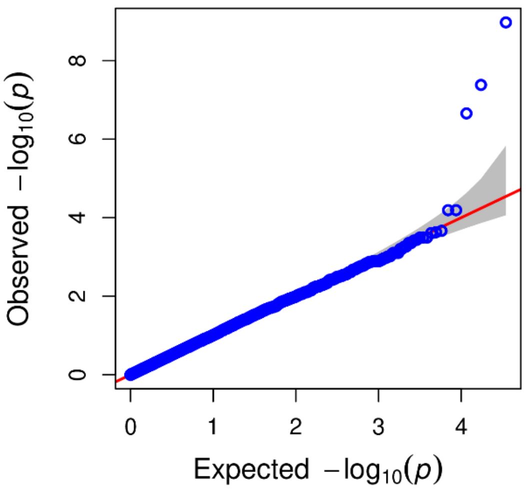

(D)

Supplementary Figure 1. Q-Q plot for two forage quality traits using 105 diverse pearl millet hybrid parents; (A) CP (crude protein) at second cut in combined analysis, (B) CP (crude protein) at second cut during summer season of 2016, (C) IVOMD (*In vitro* organic matter digestibility) at first cut during summer season 2015, and (D) IVOMD (*In vitro* organic matter digestibility) at second cut during summer season of 2016.
